# Efficacy of a personalized, response-based transdiagnostic intervention for emotional disorders delivered via the Internet: a protocol for an adaptive randomized controlled trial

**DOI:** 10.3389/fpsyt.2026.1918892

**Published:** 2026-09-21

**Authors:** Fernanda Longo Elia, Azucena García-Palacios, Cristina Botella, Juan Martín Gómez Penedo, Martin grosse Holtforth, Thomas Berger, Javier Fernández-Álvarez

**Affiliations:** 1 Fundación Aiglé, Buenos Aires, Argentina; 2 Department of Basic, Clinical Psychology and Psychobiology, Universitat Jaume I, Castellón, Spain; 3 CIBER de Physiopathology of Obesity and Nutrition (CIBEROBN), Instituto de Salud Carlos III, Madrid, Spain; 4 Vrije Universiteit Brussels, Brussels, Belgium; 5 Department of Clinical Psychology and Psychotherapy, University of Bern, Bern, Switzerland; 6 Psychosomatic Competence Center, Department of Neurology, University Hospital, Inselspital, Bern, Switzerland; 7 Department of Personality, Evaluation, and Psychological Treatments, University of Valencia, Valencia, Spain; 8 Aiglé Valencia, Valencia, Spain

**Keywords:** adaptive intervention, emotional disorders, internet based therapy, personalized treatment, transdiagnostic intervention

## Abstract

One of the main challenges in clinical psychotherapy is disseminating effective psychological treatments to the millions who need them but lack access. Transdiagnostic interventions, particularly those delivered online, can help bridge this treatment gap. However, these interventions, often provided as a “one-size-fits-all” approach, do not tailor to individual patient needs. Therefore, it is essential to identify which patients benefit from these treatments and who require more intensive interventions. This randomized controlled trial (RCT) aims to evaluate the effects of an Internet-based transdiagnostic intervention (UJI Online Transdiagnostic Protocol/Transversal) for emotional disorders, adapted to patients’ response. Three hundred sixty-six adults with clinically significant symptoms of anxiety and depression (ODSIS ≥ 7 and/or OASIS ≥ 8) and without suicidal ideation or a severe mental disorder will participate. After having completed the first three modules, patients will be randomized based on their symptomatic response. Early responders will either continue with the original intervention—12 self-administered modules—or discontinue treatment. Non-early responders will receive a blended format—including two therapist-led videoconference sessions—or continue with the original intervention. Multilevel models will be used to analyze the data. The primary expected result is that the blended format will improve treatment outcomes for non-early responders compared to the self-applied format. Moreover, it is expected that discontinuing treatment for early responders will be as effective as continuing it until completion. The findings are expected to inform clinical practice by highlighting the importance of assessing symptomatic response throughout treatment, especially in its early phases, to better tailor interventions to the needs of different patient groups.

**Clinical trial registration:**

https://clinicaltrials.gov/study/NCT07051148, identifier NCT07051148.

## 1 Background

The global demand for mental healthcare has steadily increased in recent decades. Emotional disorders (i.e., depression and anxiety disorders) are the most prevalent mental disorders, affecting approximately 4% of the world’s population at some stage in life (1). However, access to effective treatment remains limited and costly (2). As such, one of the greatest challenges for contemporary clinical psychology is the dissemination of evidence-based interventions capable of reaching millions who need them but lack adequate access.

In this context, transdiagnostic approaches have emerged as a promising solution (3). These approaches rely on treatment interventions that can be applied across a range of clinical conditions—particularly emotional disorders—by targeting shared dysfunctional psychological processes (4).

Recent meta-analyses showed the effectiveness of transdiagnostic interventions for anxiety and depression (5–7). Although these protocols enhance and expand access, not all patients benefit equally from them. Frequently delivered as “one-size-fits-all” interventions, transdiagnostic interventions present differential responses among individuals (8).

These differences are influenced by various factors, including patients’ clinical characteristics, sociocultural factors, and the relational aspects between patients and therapists (9). Consequently, it is essential to identify *what works for whom* (10) and to tailor interventions to meet the specific needs of distinct patient subgroups. The heterogeneity of treatment effects—i.e., the variability in individual responses—underscores the importance of personalization in psychotherapy (11–13).

Nye et al. (14) synthesized evidence on personalization in psychotherapy and reported a positive association between personalized interventions and improved treatment outcomes. As Cohen et al. (15) explained, personalization can be achieved by adjusting the timing, content, techniques, intensity, and delivery format of interventions. One of the advantages of personalized treatments is that they often allow for the separate testing of individual components, the adaptation of treatment sequencing and dosage for specific subgroups, and the identification of mechanisms of change that may be differentially active across patients (16, 17).

Personalization may be particularly feasible in Internet-delivered interventions, although current research on this topic remains inconclusive (18). While numerous studies have evaluated the effectiveness of online treatments (19), the question of which patients benefit most from these interventions is still underexplored. What is well established is that online psychological interventions with therapist support have demonstrated outcomes comparable to those of face-to-face cognitive-behavioral therapy (20–25).

Online interventions can be categorized into three main formats: fully self-guided without therapist assistance; self-guided with minimal human support, such as text messages, reminders, or phone calls; and blended or hybrid formats. Several studies report high dropout rates among participants in fully self-applied interventions (26). In this regard, blended formats, which incorporate individual or group synchronous sessions with a therapist, have proven to be effective alternatives to reduce attrition and enhance clinical response in Internet-based treatments, but more research is still needed to evaluate these formats (6, 27–30).

The administration of transdiagnostic treatments via the Internet has been implemented in various countries (31–35). Recent studies have demonstrated the efficacy of Internet-based transdiagnostic interventions for depression and anxiety (36). One prominent example of transdiagnostic treatment is the Unified Protocol (UP) developed by David Barlow (37, 38), which has demonstrated comparable effectiveness to diagnosis-specific treatments for disorders such as anxiety, depression, obsessive–compulsive disorder, and eating disorders (39–42). The common psychological process underlying these disorders is emotional dysregulation and its associated processes, such as distress intolerance, anxiety sensitivity, intolerance of uncertainty, experiential avoidance, and aversive reactivity (43–46).

In turn, emotion regulation has been identified as one of the principal mechanisms of change in psychotherapy in recent decades (47). It is a transtheoretical construct encompassing various dispositional and situational processes and strategies aimed at modifying one’s emotional responses to specific contexts (48, 49). Deficits in emotion regulation are implicated across a wide range of disorders and function as predisposing, triggering, and maintaining factors (50–52). Emotion regulation is a dynamic patient characteristic that can be modified, rather than the actual intervention implemented to achieve those changes (Fernández Alvarez, in preparation^1^).

To examine emotion regulation as a mechanism of change, it is necessary to investigate whether and how this process temporally precedes and influences symptomatic improvement during treatment. This entails distinguishing between stable, *trait-like* components of emotion regulation—which account for between-person variability and can predict which patients are most likely to benefit from an intervention—and dynamic, *state-like* components, which explain within-person fluctuations over the course of therapy (53).

The therapeutic alliance and patient expectations have also been extensively studied as mechanisms of change in psychotherapy. Goldfried (54) argues that fostering positive patient expectations is one of the general principles for achieving therapeutic change. In a meta-analysis that included more than 12,000 patients from 81 independent samples, Constantino et al. (55) found an association between positive patient outcome expectations and favorable treatment outcomes.

Likewise, it is well established that therapeutic alliance is a robust predictor of therapy outcomes (56, 57). However, it does not function uniformly for all patients. For some patients, it may be a facilitator that conveys the effect of another active component, while for others, with specific characteristics, it may be the active component itself. This does not depend on the theoretical orientation, but rather on the treatment design and clinical decisions (58).

### 1.1 Early response and adaptive interventions

Some studies have identified key predictors and moderators of treatment outcomes in online interventions, such as adherence, baseline symptom severity, educational level, and credibility of the treatment (59). Personalization of online interventions could increase treatment adherence (60, 61). Adherence is a critical factor in reducing dropout rates, which remain a common challenge in this format (62–65).

Among the various factors that may predict treatment response, early symptomatic change has been shown to be a reliable predictor of outcomes (66). Numerous studies have consistently demonstrated that patients who show significant symptom improvement within the first three to five sessions—referred to as *early responders*—are also more likely to achieve the most favorable outcomes (67–73). Meanwhile, meta-analyses indicate that between 30% and 40% of patients who complete a psychological treatment do not experience meaningful clinical improvement (74, 75). This highlights the necessity of adapting interventions according to patients’ clinical response, thereby distinguishing those who may benefit from low-intensity treatments from those who require more intensive modalities, such as late or *non-early responders* (76).

Collins et al. (77) distinguish between fixed and *adaptive interventions* in experimental research designs. In fixed interventions—typically tested in randomized controlled trials (RCTs)—all participants receive the same treatment, delivered in a uniform manner. In contrast, adaptive interventions incorporate decision rules that guide treatment modifications during the intervention period, based on predefined clinical criteria. These adjustments may involve changes in dosage, intensity, modality, or other aspects of the intervention to optimize outcomes (78, 79). Adaptive interventions represent one methodological approach to operationalizing personalized treatment according to patients’ clinical response. Adaptive interventions share some characteristics with stepped-care clinical models, but while stepped care primarily refers to the progressive allocation of treatment intensity in routine care, adaptive interventions constitute an experimental design for evaluating personalized treatment strategies.

In adaptive designs, randomization may not occur at the outset but rather at one or more later sequences, for example depending on the participant’s level of clinical response once the intervention is already underway (80). Such designs are particularly well-suited to evaluating the effectiveness of personalized approaches in psychotherapy, allowing researchers to tailor treatments based on individual characteristics or response trajectories.

## 2 Objectives and hypotheses

### 2.1 Objectives

The primary objective of this trial is to evaluate a personalized, response-based, transdiagnostic Internet intervention for emotional disorders in the Spanish-speaking adult population by comparing a self-applied format with minimal human support versus a blended format with synchronous videoconference therapist sessions.

The specific objectives are as follows:

1. To evaluate whether the blended format of the intervention is associated with a greater reduction in overall distress than the self-applied format among non-early responders.
2. To evaluate whether the two formats of the intervention (self-applied and blended) are associated with an improvement in state-like emotional regulation, which in turn leads to the reduction in overall distress.
3. To evaluate mediators—expectations and therapeutic alliance—in the blended condition.
4. To evaluate long-term effects at 3- and 12-month follow-up assessments.

The exploratory secondary objectives include the following:

5. To compare early self-applied responders versus non-early blended responders.
6. To compare the stop condition (3 modules) versus the continue condition (12 modules) among early responders.

### 2.2 Hypotheses

1. Non-early responders assigned to the blended condition will show a greater reduction in overall distress compared to those assigned to the self-applied condition.
2. Both intervention formats (self-applied and blended) will lead to improvements in emotional regulation, and these improvements will mediate the reduction in overall distress.
3. In the blended condition, higher treatment expectations and stronger therapeutic alliance will mediate greater reductions in overall distress.
4. Patients receiving either intervention format will maintain reductions in overall distress and improvements in emotional regulation at 3- and 12-month follow-up assessment.

Exploratory hypotheses:

5. Early responders in the self-applied condition will show comparable outcomes in overall distress reduction relative to non-early responders assigned to the blended condition.
6. Early responders who discontinue after 3 modules will show comparable outcomes to those who continue with the 12-module intervention.

## 3 Materials and methods

### 3.1 Research design

This adaptive experimental design involves a comparison of four groups. Participants are randomized after completion of module 3 (week 3), based on their early clinical response. This design allows tailoring intervention intensity *post hoc*.

### 3.2 Participants

The study will recruit a total of 366 adult Spanish-speaking participants who voluntarily express interest in participating free of charge. The patients will be recruited through the Internet Aigle Foundation website (Argentina and Valencia) and through advertising on social media. All patients will be assessed via web-based screening questionnaires, and those who fulfilled the inclusion criteria will also be assessed in a structured 30-min diagnostic video interview, also informing about the treatment setup.

Eligible participants must present with moderate to severe symptoms of depression and/or anxiety [Overall Depression Severity and Impairment Scale (ODSIS) ≥ 7 and/or Overall Anxiety Severity and Impairment Scale (OASIS) ≥ 8] and have access to the Internet and a valid email account.

The exclusion criteria include the following:

– Diagnosis of a severe mental disorder (e.g., schizophrenia or bipolar disorder)
– Current substance abuse
– Active suicidal ideation or elevated suicide risk
– Ongoing pharmacological or psychological treatment at the time of study entry
– Comorbid physical conditions that may interfere with treatment participation

Interested individuals will receive a document containing a QR code to access the Qualtrics platform, where they will review study information and provide electronic informed consent. Upon consent, participants will be assigned a unique numerical identifier to ensure anonymity and will complete the ODSIS and the OASIS as an initial screening of depressive and anxiety symptoms.

Participants who do not meet the established cutoff scores will receive an email informing them that they are not eligible for the study. Those who meet the screening criteria will be contacted by the psychologist responsible for conducting the initial diagnostic interview, who will confirm their interest in participation and their agreement with the study conditions (including potential allocation to self-guided or blended formats, online intervention delivery, and adherence to the assessment schedule).

Following this confirmation, participants who demonstrate understanding and acceptance of the study procedures will be scheduled for a videoconference session to conduct a clinical interview aimed at establishing the presence of an emotional disorder.

The therapist team—based in Argentina and Spain—will consist of clinicians from Aigle Foundation specifically trained in the UJI Online Protocol and with integrative-cognitive psychotherapy training according to the Fernández Alvarez’s integrative model (81, 82). They will have access to patients’ basic clinical information, follow a structured session guide, and participate in weekly supervision sessions with the trained supervisors leading this study.

Patients assigned to the condition that interrupts treatment at the end of module 3 will complete the intermediate assessments (weeks 6, 9, and 12) and follow-ups (at 3 and 12 months) also on the Qualtrics platform.

Recruitment of participants will follow a rolling admission process, whereby eligible participants are continuously incorporated into the trial until the total sample size is reached.

### 3.3 Sample size

Sample size calculations were performed using G*Power 3.1 (83), assuming a moderate effect size (d = 0.50), consistent with meta-analytic findings on Internet-based interventions for anxiety and depression (29). A two-tailed significance level of α  = 0.05 and a statistical power of 80% were applied.

Based on these parameters, a total of 128 participants is required within the subgroup of non-early responders (64 per condition) to ensure sufficient power to test the study’s primary hypothesis. As approximately 50% of participants are expected to be classified as non-early responders (66), the total initial sample must include 256 individuals.

To account for an anticipated dropout rate of 30%, typical of Internet-delivered interventions, the final target sample size was adjusted to 366 participants. This adjustment ensures that the statistical power of the study will be maintained even in the presence of attrition.

### 3.4 The intervention

#### 3.4.1 Self-applied format

The UJI Online Transdiagnostic Protocol is the first self-guided Internet-delivered intervention for emotional disorders developed in Spain for the adult population. It integrates the Unified Protocol modules (37) with components from dialectical behavior therapy (DBT) (84, 85). The effectiveness of this intervention has been evaluated both in community samples and within the Spanish public health system, yielding comparable results across settings: effectiveness levels similar to those of usual treatments (31, 32, 86), with superior outcomes in the reduction of depressive symptoms compared to anxiety symptoms at 12-month follow-up.

The protocol is a manualized treatment program developed by the Psychology and Technology Research Laboratory (LabPsiTec) at Jaume I University (UJI), Castellón, Spain. It is delivered via a multimedia platform designed for self-guided use by patients through the Internet.

The protocol comprises 12 modules: 11 focus on strategies to regulate negative affect, and 1 focuses on relapse prevention. Modules 1–3 cover psychoeducation, motivation for change, and the role of emotions in psychological wellbeing. Modules 4 and 5 address the acceptance and processing of emotional experiences. Modules 6 and 7 focus on cognitive flexibility training. Module 8 explores avoidance as a maintenance factor in emotional disorders. Module 9 teaches the replacement of emotion-driven maladaptive behaviors. Module 10 provides strategies for exposure to unpleasant physical sensations. Module 11 targets coping and emotional tolerance in real-life contexts. Module 12 is dedicated to relapse prevention.

Prior to implementation, the content will undergo linguistic review to ensure consistency and neutrality in the Spanish language across Spain and Latin America.

#### 3.4.2 Blended format

In the blended format, the 12 self-applied modules are supplemented by two individual 50-min sessions with a therapist delivered via videoconference. These sessions will be scheduled after module 3 and before module 9. The sessions will pursue two main goals: 1) to provide psychoeducation on the program—exploring the patient’s understanding of their symptoms and the treatment rationale, clarifying doubts, and addressing operational difficulties; and 2) to enhance adherence—discussing the pros and cons of completing tasks, meeting deadlines, and engaging with assessments.

These sessions are designed to offer personalized feedback on the benefits of continuing the intervention and to help patients connect the protocol content with their personal experiences. They are intended to support participants’ engagement with the online intervention rather than introduce additional therapeutic content beyond that provided in the self-applied modules.

#### 3.4.3 Support

CT support will consist of two weekly reminder messages delivered through WhatsApp to reinforce the importance of task completion among participants. Some examples of these messages are as follows: “Hi there! Don’t hesitate to review the modules you’ve already completed if necessary. Remember, practice makes perfect!” or “Hi there! Don’t give up on your module tasks! Dedicate some time and effort to them. Remember, it’s ideal to complete one module each week”. To minimize habituation, these messages will be randomized, so that participants do not always receive the same content. A professional platform will be used to schedule and send the messages automatically. Reminder messages are one-way only and do not allow participant responses.

## 4 Assessment

Participants will be assessed in parallel: at baseline, before and after each module, at three intermediate points (week 3, 6, and 9), at post-treatment (week 12), and at 3- and 12-month follow-ups. The assessment battery includes standardized, self-report instruments with validated adaptations for Spanish-speaking population. All outcome measures are collected through standardized self-report questionnaires completed online by participants; therefore, outcome assessment does not involve an independent evaluator.

Beyond evaluating treatment effectiveness, the assessment schedule was designed to characterize patients’ response trajectories throughout the intervention and to examine the proposed mechanisms underlying therapeutic change. The main measures are described below (**Table 1**).

**Table 1:** Assessment schedule and measures.

Assessment moment	Instrument	Assessed dimension	Number of items	Characteristics
Screening BL PM FU	Overall Anxiety Severity and Impairment Scale (OASIS; 87)	Anxiety	5	5-point Likert
Screening BL PM FU	Overall Depression Severity and Impairment Scale (ODSIS; 88)	Depression	5	5-point Likert
BL PreM (Week 3, 6, 9, 12) FU	Clinical Outcomes in Routine Evaluation (CORE-34; 89)	General distress	34	5- point Likert
BL PM (Week 3, 6, 9, 12) FU	Difficulties in Emotion Regulation Scale (DERS; 90)	Emotional regulation strategies	28	5- point Likert
BL PM (Week 3, 6, 9, 12) FU	Generalized Self-Efficacy Scale (91)	General sense of perceived self-efficacy	10	4-point Likert
BL PM (Week 3, 6, 9, 12) FU	Credibility/Expectancy Questionnaire (CEQ; 92)	Treatment outcome expectations	6	Likert 0–100%
PM	Unified Protocol Skill Use Scale (UPSUS; 93)	Emotional regulation skills	7	7-point Likert
PM	Working Alliance Inventory for Internet Interventions (WAI-I; 94)	Working alliance in internet-based interventions	12	5-point Likert
PreM (Week 1, 2, 4, 5, 7, 8, 10, 11)	CORE-10 (95)	General distress	10	5-point Likert
Week 3	System Usability Scale (SUS; 96)	Usability of a digital program or tool	10	5-point Likert
PM (Week 3, 6, 9, 12) FU	Emotional Processing Self Report (EP-SR; 97)	Emotional processing	7	5-point Likert
BL PM	Brief PANAS (98)	Positive and negative affect	10	5-point Likert
BL PM (Week 3, 6, 9, 12) FU	Inventory of Interpersonal Problems-32 (IIP-32; 99)	Interpersonal functioning	32	5-point Likert
BL PM (Week 3, 6, 9, 12) FU	Quality of Life Index (QLI-Sp; 100)	Quality of life	10	10-point Likert
BL PT (Week 3, 12)	Personality Inventory for DSM-5 (PID-5; 101)	Maladaptive personality traits proposed in the DSM-5	31	5- point Likert
BL PT (Week 3, 12)	Level of Personal Functioning Scale - Brief Form 2.0 (LPFS-BF 2.0; 102)	Levels of personality functioning according to the alternative model of personality disorders of DSM-5	12	4- point Likert

BL, Baseline; PreM, Pre-Module; PM, Post-module; PT, Post-treatment (Week 3, 12); FU, Follow-ups (3 and 12-month.)

### 4.1 Diagnostic interview

Participants who meet the inclusion criteria will be requested to complete a pretreatment clinical interview to determine the presence of an emotional disorder.

### 4.2 Clinical outcome measures

#### 4.2.1 Primary outcome measures

*Clinical Outcomes in Routine Evaluation* (CORE-34; 89). Self-report 34-item scale assessing therapeutic change in four dimensions: subjective wellbeing, problems/symptoms, general functioning, and risk. Five-point Likert scale (from 0 to 4). Adapted in Spain, Ecuador, and Chile (103–105) using the exact same version of the questionnaire.

*CORE-10* (95). Self-report 10-item questionnaire with five Likert response options. Assesses general distress. Validated for Spanish-speaking population with adequate internal consistency, convergent validity, and sensitivity to change (106).

#### 4.2.2 Secondary measures

*OASIS* (87). Self-report 5-item questionnaire that assesses frequency and severity of anxiety symptoms during the previous week. With five Likert response options (from 0 to 4). Adapted in Spain with good internal consistency (α = 0.86 ) and test–retest reliability (k = 0.84) (107).
*ODSIS* (88). Self-report 5-item questionnaire with five Likert response options (from 0 to 4). Assesses frequency and severity of depression symptoms during the previous week. Adapted in Spain with excellent psychometrics properties, internal consistency (α = 0.93), and convergent and discriminant validity (108).
Working Alliance Inventory for Internet Interventions (WAI-I; 94). A 12-item self-report questionnaire with five Likert response options. Assesses working alliance in Internet-based interventions. Validated in a German sample of adults with depressive symptoms receiving guided online intervention (94).
Difficulties in Emotion Regulation Scale (DERS; 90). Self-report 28-item questionnaire with five Likert response options. The scale assesses emotion regulation process and several difficulties associated. Adapted and validated in Spain with sound psychometric properties reported—internal consistency, test–retest reliability, and convergent and incremental validity (109).
Emotional Processing Self Report (EP-SR; 97). Self-report 7-item questionnaire with five Likert response options. Assesses emotional processing during therapeutic sessions in three independent dimensions: emotional expression, regulation, and understanding emotional meaning. The instrument demonstrates adequate psychometrics properties, with internal consistency and convergent, incremental, and predictive validity in a clinical sample. It has not yet been validated in a Spanish-speaking population.
Unified Protocol Skill Use Scale (UPSUS; 93). Self-report 7-item questionnaire with seven Likert response options. Assesses the use of emotional regulation skills from Barlow’s Unified Protocol (UP). Adapted for Spanish-speaking population (110).
Brief Positive and Negative Affect Schedule (Brief PANAS; 98). Self-report 10-item questionnaire with five Likert response options (1 to 5). Assesses positive and negative affectivity (trait/state) during the past few weeks. Adapted for Spanish-speaking population (111).
Inventory of Interpersonal Problems-32 (IIP-32; 99). Self-report 32-item scale with five Likert response options. Assesses interpersonal functioning. Validated in Spanish-speaking population with adequate internal consistency, item homogeneity, and construct and concurrent validity (112).
Quality of Life Index Spanish (QLI-Sp; 100). Self-report questionnaire containing 10 items with 10 Likert response options. Assesses physical and emotional wellbeing, social support, interpersonal functioning, personal satisfaction, and overall assessment of quality of life. Validated in Spanish-speaking population with adequate psychometric properties in a clinical sample (α = 0.87).
Generalized Self-Efficacy Scale (GSES; 91). Self-report 10-item questionnaire with a 4-point Likert scale. Assesses general sense of perceived self-efficacy. Validated in Spanish-speaking population with sound psychometric properties (113).

### 4.3 Other measures

Personality Inventory for DSM-5 (PID-5; 101). Self-report 31-item questionnaire with a 5-point Likert scale, which assesses maladaptive personality traits proposed in the DSM-5. Validated in Spanish-speaking population with adequate psychometric properties (114).

Level of Personal Functioning Scale - Brief Form 2.0 (LPFS-BF 2.0; 102). Self-report 12-item questionnaire with 4-point Likert response options. Assesses the levels of personality functioning according to the alternative model of personality disorders of DSM-5. There is a Spanish version available (115).

Credibility/Expectancy Questionnaire (CEQ; 92). Self-report questionnaire with Likert scale responses from 0% to 100%. Assesses treatment outcome expectations (patient subscale). High internal consistency (α = 0.79–0.90). Test–retest reliability: r = 0.82 for expectancy, r = 0.75 for credibility. Used in Spanish clinical trials with good results (116).

System Usability Scale (SUS; 96). Self-report 10-item questionnaire with a 5-point Likert scale (1 to 5). Good psychometric properties (α = 0.91; 117). Validated in Spain with adequate psychometric properties (118). Assesses usability of a digital program or tool (**Table 1**).

## 5 Procedure

Participants will be classified as early or non-early responders based on the Reliable Change Index (RCI), following the method proposed by Jacobson and Truax (119). This index determines whether the change in general distress scores (CORE-34) from baseline to the end of module 3 exceeds what could be expected due to measurement error, thereby qualifying as a clinically significant individual-level change.

The RCI will be calculated using the formula:

RCI = (X*post* − *X*pre) / S*diff*, where *S*diff = √2 · SD · √(1 − α)

In line with the recommendations of Lambert and Ogles (120), Cronbach’s *α* coefficients of internal consistency will be used instead of test–retest reliability estimates, since *α* provides a more stable and widely available indicator of measurement precision. Standard deviations and *α* coefficients from Spanish validation studies will be applied (103, 105). A reliable change will be defined as one that equals or exceeds ±1.96 × S_diff (*p* < 0.05, two-tailed). Participants exceeding this threshold will be considered early responders; those below it will be classified as non-early responders (**Figure 1**).

**Figure 1:**
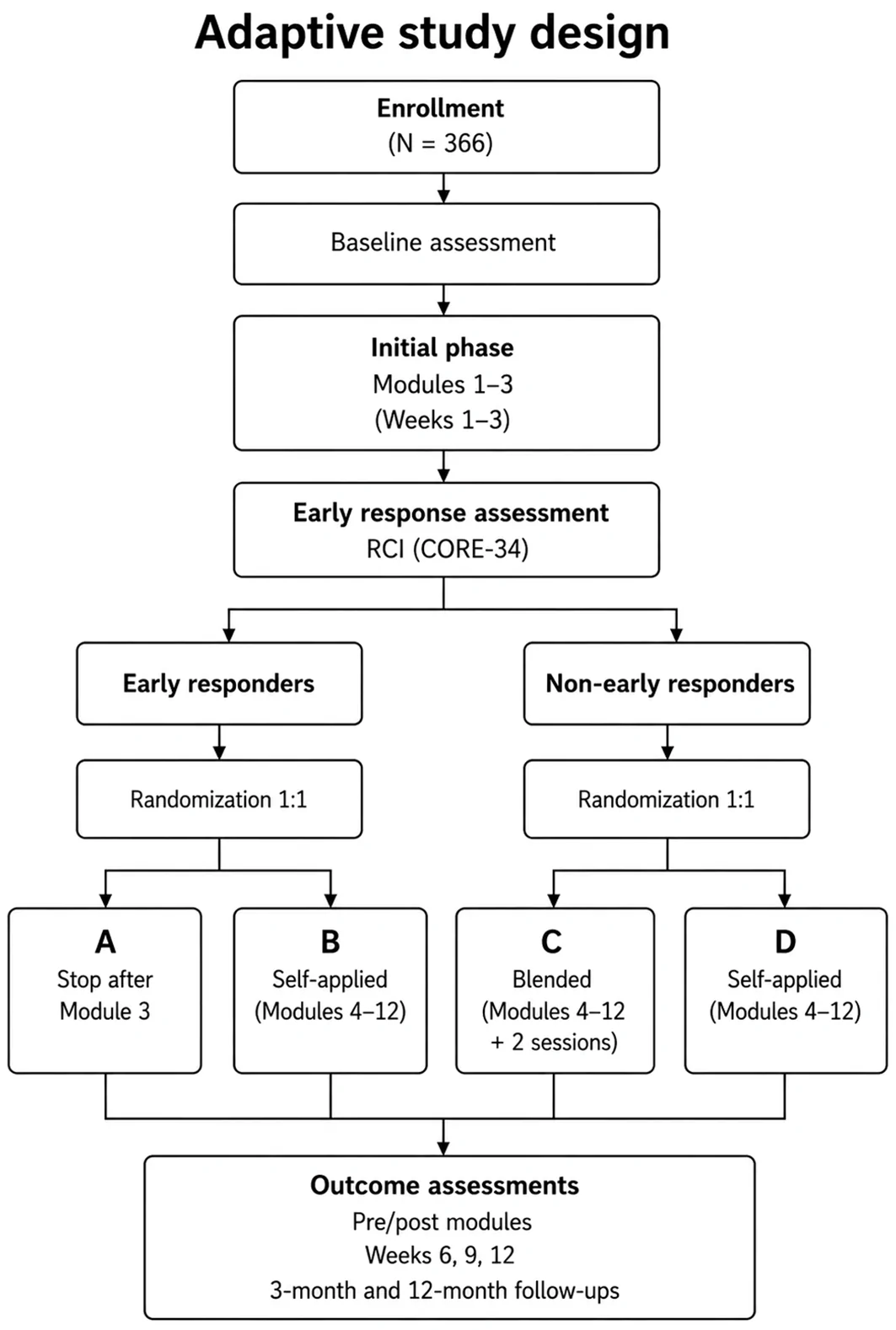
Adaptive study design. ER, early responders; NER, non-early responders; RCI, Reliable Change Index.

All participants will begin the intervention by completing the first three modules of the protocol. The intervention is designed to be completed at a pace of one module per week; accordingly, completion of module 3 is expected to occur around week 3 (**Figure 2**). Randomization is performed after each participant completes module 3. This module was selected as the study decision point because it marks the completion of the initial phase of the intervention, during which participants receive the core psychoeducational and motivational components that are common to most psychotherapeutic approaches, before progressing to the intervention-specific therapeutic strategies. This timing was considered sufficient to detect early symptomatic change while still allowing treatment intensity to be modified during the remainder of the intervention.

**Figure 2:**
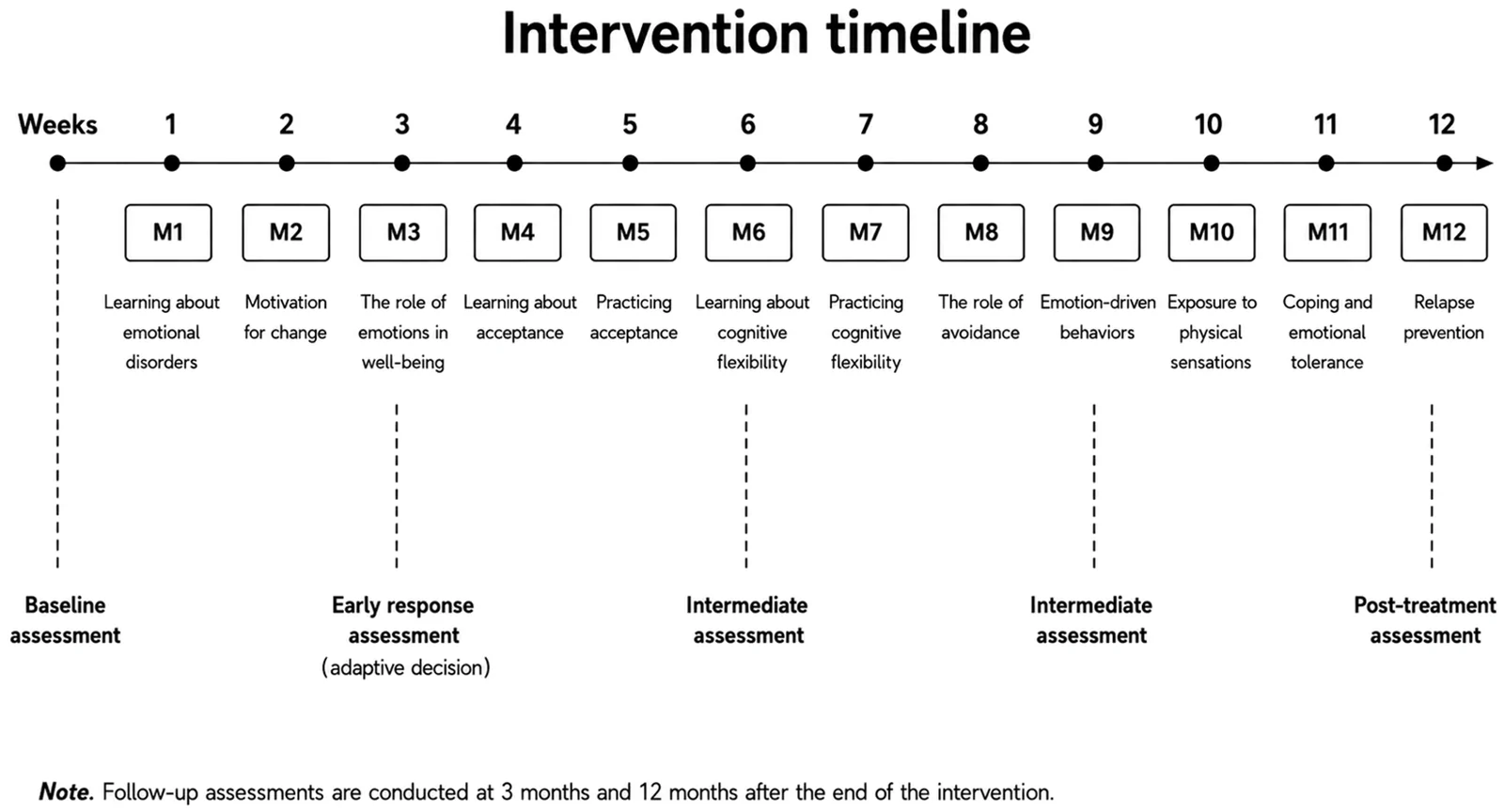
Intervention timeline.

After completing module 3, participants’ clinical response will be assessed. Randomization will occur only after this assessment and separately within each response group. Participants classified as early responders will be randomly assigned to one of the following two conditions:

A) Discontinue treatment after module 3.

B) Continue with the self-applied intervention until completion.

Participants classified as non-early responders will be randomly assigned to one of the following two conditions:

C) Receive the blended intervention (including two individual sessions with a therapist between modules 3 and 9).

D) Continue with the self-applied intervention until completion.

Repeated outcome assessments provide ongoing clinical monitoring throughout the intervention, allowing early identification of symptom worsening or emerging clinical risk. In addition, the platform incorporates an automated alert system for suicidal ideation based on participants’ questionnaire responses. If an alert is triggered, the clinician responsible for participant follow-up will immediately activate the study’s predefined risk management protocol, including direct clinical contact. Participants identified as being at risk of suicide will be immediately withdrawn from the study intervention and referred for appropriate in-person clinical care.

## 6 Randomization

Simple randomization will be performed using a 1:1 allocation ratio to assign participants to their respective experimental conditions. Randomization will be conducted using a computerized algorithm based on a randomly generated sequence (‘randomizeR,’ ‘blockrand,’ or ‘sample()’ function in R base) (121).

The allocation sequence will be managed by an external researcher who is blinded to the clinical team. This researcher will use password-protected, preloaded randomization lists to ensure allocation concealment. No stratification by sex, age, or socioeconomic status will be applied. Assignment will occur individually and in real time within each stratum, without blocking or other restrictions, thereby ensuring numerical balance across conditions as recruitment progresses.

## 7 Statistical analyses

Primary outcomes will be analyzed using multilevel models. The main dependent variable is the reduction in general distress, measured with CORE-34 scores at baseline; weeks 3, 6, 9, and 12; and at 3- and 12-month follow-ups. Repeated measures will be nested within participants, and condition-by-time interactions will be used to estimate differential trajectories of symptom change across groups.

Reliable deterioration will be examined as a secondary safety outcome and defined as an increase in CORE-34 scores from baseline to post-treatment exceeding the reliable change threshold in the direction of worsening. The threshold will be calculated using the same Reliable Change Index methodology described above. Rates will be reported by study condition. This individual-level classification will complement the multilevel analyses of continuous symptom trajectories.

Moderation analyses will be conducted to examine the impact of baseline severity, expectations, and therapeutic alliance, using multilevel models. Mediation analyses will be used to explore mechanisms of change—emotional regulation, alliance, expectations—via longitudinal structural equation modeling with bootstrap estimation of non-parametric confidence intervals for indirect effects. Therapist-level variability will be explored by considering therapist as an additional level in the multilevel models.

Additional sensitivity analyses will be performed to assess the impact of missing data. Effect sizes (Cohen’s *d*, marginal and conditional *R*^2^) will be reported for all relevant comparisons. Correction for multiple comparisons will be applied in the secondary analyses.

Statistical analyses will be conducted by a researcher blinded to treatment allocation. Intervention groups will remain coded until completion of the primary analyses. All analyses will adhere to the intention-to-treat (ITT) principle, whereby all participants will be included in the analysis regardless of their adherence level to mitigate selection bias due to dropout.

## 8 Discussion

This study represents an original methodological contribution: it is the first randomized controlled trial to evaluate an adaptive Internet-delivered transdiagnostic intervention for emotional disorders, personalized according to patients’ clinical response. The study seeks to identify which subgroups of patients can benefit from a self-applied format and which may require a higher-intensity (blended) format.

To date, no studies worldwide have empirically tested this question, despite its high clinical relevance. The findings are expected to contribute to the optimization of scalable mental health interventions by providing evidence to guide intensity-based treatment decisions. This trial also offers a valuable opportunity to assess the feasibility, effectiveness, and mechanisms of change associated with personalization strategies.

In line with the study’s main hypothesis, it is expected that among patients who exhibit a delayed response to treatment, those assigned to the blended condition will show greater improvements in general distress compared to those who continue with the self-applied standard format. Such findings would provide important empirical support for the personalization of psychological interventions based on patient characteristics and response patterns (14). Identify which patients can benefit from a self-applied format and who may require a higher intensity treatment could save time, money, and suffering for patients.

We also expect to clarify the role of emotional regulation as the primary mechanism of change predicting treatment outcomes. Furthermore, we assume that patients’ outcome expectations and the therapeutic alliance (both with the program itself and with the therapist, in the case of blended condition) may also be mediating variables in treatment outcomes, especially for non-responder patients or those with a delayed response at the start of the intervention.

In addition, if the exploratory hypothesis is confirmed, namely, that a reduced dose of the self-applied protocol (three modules) is as effective as the full version for early responders—this would have significant implications for clinical practice. Specifically, it would challenge traditional assumptions about the necessary intensity and duration of treatment, encouraging more cost-effective and personalized care pathways in mental health services.

## 9 Limitations

One potential limitation of this study is the minimal therapist involvement in the blended condition, which includes only two synchronous sessions. While this design favors scalability and cost-effectiveness, it may reduce the extent to which therapeutic alliance and individualized support can impact outcomes. Future studies should compare blended interventions with usual treatment, involving more therapist synchronous sessions and assessing its effectiveness under routine care conditions.

Additionally, this research does not include prospective algorithms for assigning patients to treatment formats based on pretreatment characteristics. Future developments should explore the use of predictive analytics and stepped-care algorithms to optimize treatment allocation from the outset and enhance the personalization of digital mental health interventions.

Finally, because the intervention is delivered entirely online, the study sample may not be fully representative of the broader population seeking psychological treatment. Participation requires access to the Internet and familiarity with digital technologies, which may reduce the inclusion of individuals with limited access to or confidence in using online platforms. Consequently, the generalizability of the findings to these populations should be interpreted with caution.

## 10 Ethical considerations

This study has been registered on ClinicalTrials.gov (ref Nr. NCT07051148) and approved by the Human Research Ethics of the University Jaume I of Castelló de la Plana (UJI), Spain (file CEISH/56/25). All participants will provide written informed consent prior to beginning the intervention. They will not be informed of their assigned experimental condition in advance and may withdraw from the study at any time or if they no longer meet the inclusion criteria. Patients were not involved in the design, conduct, reporting, or dissemination plans of this research. Participants will be recruited after the study design was finalized.

All data collected during the study will be handled in accordance with applicable data protection regulations to ensure participant confidentiality. The protocol adheres to the CONSORT-EHEALTH guidelines (Consolidated Standards of Reporting Trials of Electronic and Mobile Health Applications and Online Telehealth) and the Adaptive Designs CONSORT Extension (ACE) to ensure transparency and methodological rigor in reporting.

## Footnotes

1 Fernández-Álvarez J, Babl A, Gómez Penedo JM, García-Palacios A, Grosse Holtforth M. Emotion regulation as a transtheoretical and transdiagnostic factor in psychotherapy: challenges and pathways forward.
